# Current status and future promise of next-generation poly (ADP-Ribose) polymerase 1-selective inhibitor AZD5305

**DOI:** 10.3389/fphar.2022.979873

**Published:** 2023-01-23

**Authors:** Jingcao Zheng, Zhengyu Li, Wenjiao Min

**Affiliations:** ^1^ Department of Gynecology and Obstetrics, West China Second University Hospital, Sichuan University, Chengdu, China; ^2^ Key Laboratory of Birth Defects and Related Diseases of Women and Children, Sichuan University, Ministry of Education, Chengdu, China; ^3^ Psychosomatic Department, Sichuan Provincial People’s Hospital, University of Electronic Science and Technology of China, Chinese Academy of Sciences Sichuan Translational Medicine Research Hospital, Chengdu, China

**Keywords:** PARP inhibitors, improved selectivity, novel therapy, oncology, AZD5305

## Abstract

The family of poly (ADP-ribose) polymerases (PARPs) consists of 17 members, which have been demonstrated as having effects on a series of cellular processes, including DNA replication and repair. PARP inhibitors (PARPi) suppress DNA repair through “PARP trapping”, thus, constitute an important treatment option for cancer nowadays. In addition, PARP inhibition and homologous recombination repair (HRR) defects are synthetically lethal, giving a promising therapeutic for homologous recombination repair deficient (HRD) tumors including BRCA mutation. However, overlapping hematologic toxicity causes PARPi to fail in combination with some first-line chemotherapies. Furthermore, recent literature has demonstrated that PARP1 inhibition and PARP1-DNA trapping are key for antitumor activity in HRD cancer models. Currently approved PARPi have shown varying levels of selectivity for the entire 17-member PARP family, hence contribute to toxicity. Together, these findings above have provided the necessity and feasibility of developing next-generation PARPi with improved selectivity for PARP1, expanding significant clinical values and wide application prospects both in monotherapy and combination with other anticancer agents. In this review, we summery the latest research of current approved PARPi, discuss the current status and future promise of next-generation PARP1-selective inhibitor AZD5305, including its reported progress up to now and anticipated impact on clinical.

## 1 Introduction

In the last 50 years ago, since poly (ADP-ribose) polymerase 1 (PARP1) was first discovered (Nishizuka et al., 1967), the field of PARP biology has expanded dramatically. Advances in our understanding of the role of PARP1 in DNA repair have expanded the clinical application of PARP inhibitors in various diseases, especially cancers, as tumor cells are highly sensitive to PARP inhibition because of DNA repair defects (Curtin and Szabo, 2020). For example, germline or somatic aberrations in the DNA damage repair genes are found in 19% of primary prostate cancer and almost 23% of metastatic castration-resistant prostate cancer (Boussios et al., 2021). Among the 17 members of PARP family, PARP1 is the best described enzyme and plays a critical role in the repair of single strand breaks (SSB) through the base excision repair (BER) pathway. Furthermore, PARP2 has been implicated in the regulation of red blood cell production (erythropoiesis) (Farrés et al., 2015). This review provides a brief summary about mechanisms of PARP inhibition in oncology, introduces the adverse effects of approved PARPi, more importantly, provides current status and future promise of next-generation PARP 1-selective inhibitor AZD5305.

## 2 PARP inhibition in oncology

PARPi with the capacity for PARP1 and PARP2 (PARP1/2) DNA trapping, have achieved widespread success as a target therapeutics for a variety of cancers, as ovarian cancer, breast cancer, pancreatic cancer and prostate cancer in particular, both in monotherapy and in combination with additional therapies such as anti-angiogenic agents or immune checkpoint inhibitors (Gong et al., 2020a). Molecular mechanisms of the anticancer effects including “PARP trapping” and synthetically lethal.

### 2.1 PARP inhibitors suppress DNA repair through “PARP trapping”

SSBs caused by chemical or physical interference, such as chemical reagents and ionizing radiation, may be repaired by PARP pathway, through the mechanism of PARP1/2 bind to DNA breaks and recruit repair enzymes *via* auto-PARylation, resulting in tumor cell survival. PARPi suppress DNA repair through “PARP trapping” (Curtin and Szabo, 2020) [a term coined to refer to that under the application of PARPi, the prevention of PARP1 dissociation from the DNA (Kedar et al., 2012; Pommier et al., 2016)], inhibit the catalytic activity and prevent auto-PARylation, thus increase the number of DNA damage lesions and cause cell death (Pommier et al., 2016; Zandarashvili et al., 2020).

### 2.2 PARP inhibition and HRR defects are synthetically lethal

SSBs accumulate and cause replication fork collapse when PARP is inhibited, thus, lead to the DNA double-strand breaks (DNA DSBs) with genotoxicity (Farmer et al., 2005). Under the circumstance, HRR will be induced, proteins like BRCA1 and BRCA2 play a role in signalling and repairing between cells. However, when lacking HRR function (HRD), as in BRCA-mutant (BRCAm) cells, DNA DSBs will be processed by alternative but error-prone repair pathways (NHEJ, non-homologous end joining repair), which lead to the accumulation of genomic instability and ultimately cancer cell death (Bryant et al., 2005; Farmer et al., 2005).

## 3 Current approved PARP inhibitors

In 2014, olaparib was approved (Kim et al., 2015), followed by rucaparib in 2016 (Balasubramaniam et al., 2017), niraparib in 2017 (Ison et al., 2018), and talazoparib in 2018 (Hoy, 2018). Veliparib has not yet received regulatory approval, as further clinical research is still ongoing (Murai et al., 2014). Talazoparib with the lowest approved efficacious dosage is considered as the most potent clinically used PARPi (Curtin and Szabo, 2020). It is noteworthy that these approved PARPi were all initially discovered neither after the concept of PARP1-DNA trapping, nor the theory of synthetic lethality (Johannes et al., 2021).

Obviously, BRCAm cancer patients remain the main beneficiaries, however, many recent clinical trials have found that the non-BRCAm cancer patients can indeed gain survival benefits from PARPi, indicating the expanded application of PARPi, emphasizing the value of molecular characterization for cancer treatment decisions. Therefore, exploring a reliable and unified biomarker detection method to screen patients and select appropriate PARPi is demanded (Xu and Li, 2021). In the phase IIIb OPINION trial (NCT03402841) (Poveda et al., 2022), maintenance olaparib demonstrated clinical benefit in patients without a germline BRCA1/BRCA2 mutation (gBRCAm). Additionally, the phase Ⅲ trial L-MOCA (NCT03534453) (Gao et al., 2022), focused on olaparib maintenance monotherapy in Asian patients with platinum-sensitive relapsed (PSR) ovarian cancer has been published this year. As the first study to aim at the efficacy and tolerability of olaparib that specifically for Asian patients with PSR ovarian cancer, it highlights the promising efficacy of olaparib in Asian population. This open-label, single-arm, phase III study enrolled patients from countrywide clinical centers across China and Malaysia with high grade epithelial PSR ovarian cancer. Results suggested that regardless of BRCA status, olaparib maintenance therapy was relatively effective and well tolerated in Asian patients with PSR ovarian cancer. Likewise, the OReO study (NCT03106987), a phase IIIb, randomized, double-blind, placebo-controlled, multicenter study, is the first study to assess and further demonstrate the advanced efficacy and tolerability of olaparib retreatment in patients with PSR ovarian cancer, regardless of BRCA status. The new results of OReO study were presented at the 2021 Congress of the European Society of Medical Oncology (ESMO 2021). Apart from olaparib, a recent study has brought a new era of niraparib in first-line maintenance therapy of ovarian cancer in China. The PRIME trial (NCT03709316), a randomized, double-blind, placebo-controlled, multi-center, phase III clinical trial, enrolled advanced ovarian cancer patients who have achieved effective response after first-line platinum-containing chemotherapy, with the purpose of evaluating the safety and efficacy of ZL-2306 (niraparib) for maintenance treatment, has obtained positive results. As the first largest clinical trial used PARPi in maintenance therapy for advanced first-line ovarian cancer patients in China, as well as the first phase Ⅲ clinical study in the world to prospectively use individualized starting dose in treatment, its exciting data were presented on the Society of Gynecological Oncology (SGO) 2022 Annual Meeting. Notably, niraparib has been included in the 2022 Catalog of Medicines Covered by National Medical Insurance System in China. Apart from ovarian cancer, a latest phase II study (NCT02032823) has demonstrated that PARP inhibition is also an effective treatment for patients with metastatic breast cancer with gPALB2 (ORR, 82%) and sBRCA1/2 (ORR, 50%) mutations, significantly expanding the population of breast cancer patients who may benefit from PARPi, indicating that not only gBRCA1/2 mutation carriers, but also other mutations in homologous recombination-related genes may benefit from PARPi (Tung et al., 2020). Likewise, according to an article published in the Lancet Oncology July 2022 (NCT03404960), PARPi combined with nivolumab or niraparib has shown effectiveness among pancreatic cancer patients, even though the BRCA mutation rate in pancreatic cancer is only about 4%–7% (Reiss et al., 2022). Studies above suggested that regardless of biomarker status, PARPi may still show clinical benefits, indicating the application of PARPi in the treatment of non-BRCAm cancer is expected.

### 3.1 Adverse effects

The specific mechanism of PARPi is to target cancer cells based on their inherent deficiencies, while appearing to avoid normally functioning cells, thus, may infer a low toxicity profile. However, these first-generation drugs inhibit both PARP1 and PARP2 (as well as other PARP family members) and generally present a class of undesirable adverse event, including hematological effects, gastrointestinal effects, renal toxicities, fatigue and so on (Dellavedova et al., 2021). These adverse effects common to PARPi are usually mild, tend to occur during the early stage of treatment and are managed by dose interruption or dose reduction, but they can be serious in rare cases, leading to dose discontinuation. It is worth noting that the performance of PARPi declines due to increased risks of selected adverse events, moreover, side effects have limited the clinical applicability of PARPi, especially in combination with already poorly tolerated chemotherapeutic agents (Dellavedova et al., 2021). Additionally, not only from the efficacy and toxicity aspects, combining PARPi in therapeutic is not currently recommended from the cost-effective perspective (Gong et al., 2020b). Notably, PARP2 is particularly linked with the hematological toxicities, which arouse the interest of developing next-generation PARPi with improved selectivity for PARP1. Summary of adverse events in phase Ⅲ trials of PARPi in the front-line ovarian/breast cancer setting is shown in Supplementary Materials.

#### 3.1.1 Hematological toxicities

Hematological toxicities are a very common class effect of PARPi, which tend to occur early after treatment initiation with recovery several months later, sometimes requiring dose interuptions or reductions (Berek et al., 2018). According to the six phase Ⅲ trials in the front-line ovarian cancer setting (Supplementary Table S1) (Mirza et al., 2016; Coleman et al., 2017; Pujade-Lauraine et al., 2017; Moore et al., 2018; González-Martín et al., 2019; Ray-Coquard et al., 2019), as well as in the three phase Ⅲ trials in the front-line breast cancer setting (Supplementary Table S2) (Robson et al., 2017; Litton et al., 2018; Tutt et al., 2021), anemia is the most common hematological disorders among PARPi, other common hematologic events including thrombocytopenia, neutropenia and leukopenia. A systematic review analyzing relevant clinical trials and comprising 4,553 patients with advanced ovarian cancer for evaluation of toxicity profile showed that patients treated with PARPi exhibited higher risks of all-grade and high-grade hematological toxicities, including anemia, leucopenia, neutropenia and thrombocytopenia (Hao et al., 2021). Mainly due to the bone marrow toxicity and negative effects on the peripheral blood, anemia notably is the most common adverse event reported from dual PARP1/2 inhibitors, as PARP2 having been particularly related to erythropoiesis (Farrés et al., 2015). Observed hematological disorders such as anemia, thrombocytopenia and neutropenia are both more pronounced with niraparib comparing with rucaparib and olaparib, therefore, a weekly testing in the first month of platelet concentrations is recommended by the niraparib FDA label (ZEJULATM (niraparib) capsules, 2019). It has been demonstrated that the cause of thrombocytopenia with the applying of PARPi is associated with a reversible decrease in megakaryocyte proliferation and maturation (Berek et al., 2018). In order to monitor hematological toxicity, an intermittent complete blood count is recommended for patients starting treated with PARPi or those who undergo a dose modification.

#### 3.1.2 Gastrointestinal toxicities

Based on six ovarian cancer studies (Mirza et al., 2016; Coleman et al., 2017; Pujade-Lauraine et al., 2017; Moore et al., 2018; González-Martín et al., 2019; Ray-Coquard et al., 2019) and three breast cancer studies (Robson et al., 2017; Litton et al., 2018; Tutt et al., 2021), nausea is most common among all the gastrointestinal adverse events, which is a common class effect adverse event for PARPi occuring at an early stage and is considered to be worse during the first cycles (Madariaga et al., 2020). Other frequent gastrointestinal symptoms include constipation, vomiting, decreased appetite, abdominal pain and diarrhea. A meta-analysis included 29 randomized controlled trials (RCTs) and 9,529 patients suggested that patients who received veliparib had a relatively lower risk of all-grade vomiting and nausea (Sun et al., 2021). Evaluation or monitoring of weight loss might be helpful, as for the treatments, nutrition consultation is recommended (National Comprehensive Cancer Network (NCCN), 2019), and medication as prokinetic and anti-histamine drugs are generally helpful to meliorate symptoms.

#### 3.1.3 Renal toxicities

Increased creatinine concentration is another common adverse event in the presence of PARPi, mainly due to the interacts with MATE1 and MATE2-K renal transporter proteins, which play a important role in renal secretion (LaFargue et al., 2019). Importantly, elevation of serum creatinine not necessarily reflect to kidney insufficiency, thus a thorough assessment of glomerular filtration rate (GFR) is recommended when concern exists for renal disfunction.

#### 3.1.4 General toxicities

General disorders include fatigue/asthenia, a universal toxicity for nearly all PARPi, which also captured in high frequency by the patient-reported outcomes (Oza et al., 2018). Experts recommend that non-pharmacological treatments, such as exercise and cognitive behavioral therapy to reduce symptoms.

#### 3.1.5 Less common toxicities

The incidence rate of rare adverse events continues to increase, especially with some of the novel combination regimens. Some of the more common, yet overall infrequent, however cannot be entirely ignored toxicities of PARPi affect the neurological, respiratory, psychiatric, musculoskeletal, and connective tissue, cardiovascular systems, for example, myelodysplastic syndrome is a rare but serious adverse event related to PARPi treatment, found in approximately 1% of patients (ZEJULATM (niraparib) capsules, 2019), (LYNPARZA^®^ (olaparib). U.S, 2019; RUBRACA^®^ (rucaparib) tablets, 2019).

### 3.2 The development of PARP inhibitor resistance

PARPi resistance is ubiquitous in clinic. It is reported that over 40% of BRCAm ovarian cancer patients failed to benefit from PARPi (Audeh et al., 2010; Fong et al., 2010). As HRD is the main prerequisite for the anticancer effects of PARPi, HRR becomes the predominant cause of PARPi resistance. It is suggested that PARPi resistance can occur through several different mechanisms: restoration of HRR pathway, protection of the DNA replication fork, increasing PARylation activity, reversion mutations, epigenetic modification and pharmacological alteration (Li et al., 2020).

In order to overcome PARPi resistance, the optimal combination of PARPi with other therapeutic regimens are urgently required. Combinatorial synergy and patient benefit may be described in the currently ongoing clinical trials with different agents. Yet, it is undeniable that overlapping hematologic toxicities from different targeted therapy with the current approved PARPi is a major limiting factor in the design. We assumed that the high selectivity of next-generation PARP1 inhibitor may potentially allow more efficacious doses of partner therapies to be applied in combination. Thus, much work is urgently needed to comprehensively understand PARPi resistance mechanisms and effectively promote novel PARPi.

## 4 PARP1-selective inhibitor AZD5305

Studies by Murai et al. (2012) and Ronson et al. (2018) have both demonstrated that loss of PARP1 is the major driver of synthetic lethality with HRD, that is, the trapping of PARP2 to DNA is actually not necessary for synthetic lethality. Along with the increased risks of selected adverse events and the development of PARPi resistance, an exploration of next-generation PARPi with improved selectivity for PARP1 is desperately needed. Fortunately, a latest published literature delivered a novel, highly selective and potent PARP1 inhibitor AZD5305 (Johannes et al., 2021), with its good pharmacokinetics (PK), pharmacodynamics (PD) and safety pharmacology *in vivo* model, this compound represents a next-generation PARPi. These surprising findings were presented during the American Association for Cancer Research Annual Meeting 2022 in New Orleans, LA, held April 8–13 (AZD5305 More Tolerable than Earlier, 2022).

### 4.1 Discovery process

NMS-P118 is the first PARP1 selective inhibitor with demonstrated anticancer activity both in monotherapy and in combination (Papeo et al., 2015). Inspired by this work, Johannes et al. (2021) screened an extensive characterization of previously reported PARPi and initiated a medicinal chemistry program building on the high selectivity of FR257531. Using parallel chemistry to generate diverse analogs, X-ray crystallography to enable structure-based design, and with exploration of multiple nicotinamide mimetic cores, researchers were able to generate lead compound AZ4554, after made key improvements in secondary pharmacology including solving the hERG issues and reducing logD, subsequently obtained AZD5305.

### 4.2 Pharmacokinetics (PK) and Pharmacodynamics (PD)

AZD5305 exhibited a very favorable preclinical PK profile, very low plasma clearances, generally low steady-state volumes of distribution and high oral bioavailability in a preclinical investigation *in vivo* (Johannes et al., 2021). Along with its good potency, AZD5305 was expected to drive low efficacious doses, reflecting its high therapeutic potential. Another investigation of the PK/PD relationship in BRCA1m triple-negative breast cancer (TNBC) xenograft model MDA-MB-436 indicated that the maximum efficacy of AZD5305 was achieved when unbound plasma concentrations were maintained above the IC95 (Staniszewska et al., 2021).

### 4.3 Selectivity

It is found that AZD5305 is not only having the desired and promising PARP1 selectivity profile (∼500-fold vs. PARP2) but also has a surprising clean profile across the entire PARP family (Johannes et al., 2021), revealing its high selectivity. An immunofluorescence-based assay provided further proof, as even the tested concentrations were as low as nanomolar (nM), AZD5305 was able to selectively induce PARP1 trapping, whereas PARP2-trapping was not observed under any of the conditions (Illuzzi et al., 2021). These features lead to potent antiproliferative effects in HRR-deficient cells and minimal/no effects in HRR-proficient cells (Illuzzi et al., 2022). It is unprecedented that AZD5305 is able to specifically target cancer cell models with HRR-deficiency, while sparing HRR-proficient cells (Pilie et al., 2019).

Understanding the width of the therapeutic window for a novel drug is essential for developing effective and safe treatment plans. AZD5305 induced DNA damage and cell death on BRCAm cells, for example, the PALB2- and RAD51-mutated cell lines, with antiproliferative IC50 in the single-digit nM, whilst having minimal effects on isogenic BRCA-wild-type cell (Illuzzi et al., 2021). This was in contrast to the first-generation PARPi, which induced non-selective DNA damage. To observe the difference of effects on DNA repair deficient and proficient cell lines, indicates the wide therapeutic window and great potential of AZD5305 in clinical applications. Finally, the secondary pharmacology of AZD5305 is remarkably clean, showing high selectivity against a group of pharmacology targets, as only 8 (PDE6, PDE2A, PDE3A, dopamine receptor D4, ACHE, adenosine receptor A1, PDE10A2) of 98 targets tested had IC50 < 30 μM (Hande et al., 2021), indicating its less off-target activity.

Screening of AZD5305 and other PARPi on a panel of larger cell lines indicated that, as treated with different sensitivity cells, a differential clustering was revealed, which suggested that in different genetic backgrounds, AZD5305 may be utilized as a unique tool to explore and refine selective PARP1-related activities in cancer cells, and the effects of targeting them (Illuzzi et al., 2021). With this goal, Illuzzi et al. (2021) are currently performing CRISPR/Cas9 screens in order to identify genes that cause sensitization to AZD5305 when downregulation.

### 4.4 Potency

AZD5305 was tested for efficacy in BRCA1m TNBC patient-derived explant (PDX) model HBCx17, MDA-MB-436 model, DLD-1 BRCA2−/− and wild-type isogenic xenograft model respectively (Staniszewska et al., 2021). Similar results were obtained in the first three models, as it showed excellent potency compared with olaparib, enabled anti-tumor effects and tumor regression under a low-level dosage. Importantly, anti-tumor effects of AZD5305 continued after treatment withdrawal and sustained over 100 days, in contrast to the olaparib-treated group where regrowth of tumors was observed from day 63. As expected, AZD5305 showed no efficacy in the last model, again confirming its selectivity for HRD cells. Moreover, an investigation in BRCA1m TNBC xenograft model SUM149PT and BRCA WT TNBC PDX model HBCx-9 indicated that, comparing to each monotherapy treatment, AZD5305 was well tolerated and showed significant benefit when combined with carboplatin (Staniszewska et al., 2021). Likewise, another investigation in ovarian cancer xenografts (OC-PDXs) model had come to a conclusion that combined with carboplatin, AZD5305 stabilized the growth of tumours at doses as low as .1 mg/kg that on its own was not effective (Dellavedova et al., 2021). To sum up, AZD5305 showed improved efficacy and tolerability as monotherapy and in combination with standard of care chemotherapy in the *in vivo* preclinical models when compared to non-selective PARPi.

### 4.5 Hematologic toxicity

An hematotoxicity assay suggested that AZD5305 exerts less toxicity on human *in vitro* hematopoietic stem/progenitor cells (HSPCs) than talazoparib (Johannes et al., 2021). In rat models as a monotherapy, in comparison with olaparib, AZD5305 does not cause hematological toxicity at predicted clinically efficacious exposures, however, olaparib causes up to 50% reduction in hemoglobin (Leo and Johannes, 2021), again demonstrating that monotherapy toxicity of PARP1/2 inhibitors may be dependent on PARP2 inhibition. Furthermore, according to a subsequent rat *in vivo* study of combination chemotherapy, AZD5305 + carboplatin showed improved serologic tolerance compared with olaparib + carboplatin, as peripheral reticulocytes and myeloid erythroid precursor cells recover with continuous AZD5305 administration but not in the presence of continuous olaparib (Gill et al., 2021). Notably, this differentiation was maintained and confirmed again in a more closely mimic clinical protocol with two-weekly cycles and higher dose of carboplatin (Gill et al., 2021). Thus, regardless of monotherapy or in carboplatin combinations, AZD5305 has improved hematological tolerability over dual PARP1/2 inhibitors in rodents (Gill et al., 2021). Findings above has confirmed the pathogenic role of PARP2-associated hematologic toxicity, thus targeting only PARP1 can retain the therapeutic benefit of non-selective PARPi, while reducing potential for hematotoxicity (Illuzzi et al., 2022). Notably, provided factual evidence and further rationale for the improved hematologic profile expected for AZD5305 both in monotherapy and combination chemotherapy. We firmly believe that AZD5305 could overtake the current approved PARPi in combination strategy development, in particular, when the partner drug is prone to have hematologic toxicity.

### 4.6 Clinical development

In consideration of its impressive selectivity, enhanced potency, and improved toxicity profile in preclinical studies, AZD5305 is a promising next-generation PARPi and has been progressed into a first-in-human phase I/IIa modular, open-label, multi-center clinical trial on 12 November 2020 (NCT04644068, AstraZeneca, Cambridge, United Kingdom) (Supplementary Table S3). This research is designed to assess the safety, tolerability, pharmacokinetics, pharmacodynamics and preliminary efficacy of ascending doses of AZD5305 administered orally, either as monotherapy or in combination with anti-cancer agents in patients with advanced solid malignancies. The study is estimated to enroll 715 participants (age 18–130) and consists of five individual intervention modules, each evaluating the tolerability and safety of AZD5305 dosed both as monotherapy or in combination with a specific partner, involving paclitaxel (Ⅳ anti-microtubule agent), carboplatin (Ⅳ platinum chemotherapeutic), T-DXd and Dato-DXd (Ⅳ antibody-drug conjugate). The specific combination partner of the 2–5 modules is paclitaxel, carboplatin with or without paclitaxel, T DXd, Dato-DXd respectively. The control group (module 1) has only AZD5305 monotherapy. Each module is made up of two study parts (part A/B), consisting of dose-escalation and expansion cohorts respectively. Module 4 and 5 have only part A. Importantly, patients in part B must not have received prior PARPi-related therapy, either as a treatment or as maintenance. Detailed information about the studied diseases is ovarian cancer, breast cancer, pancreatic cancer, prostate cancer, and several additional indications for module 4 and 5, which include colorectal cancer, non-small cell lung cancer, biliary cancer, small cell lung cancer, bladder cancer, gastric cancer, cervical cancer, and endometrial cancer. Primary outcome measures contain the number of subjects with adverse events/serious adverse events, and the number of subjects with dose-limiting toxicity (DLT). The study started on 12 November 2020, approximately complete on 29 July 2025.

A newly launched study named PETRANHA was designed to evaluate the safety, tolerability, pharmacokinetics, pharmacodynamics and preliminary efficacy of AZD5305 when given in combination with new hormonal agents (enzalutamide, abiraterone acetate, darolutamide) in patients with metastatic prostate cancer (Supplementary Table S3). This is a multi-arm, open-label phase I/IIa study that estimated to be completed on 12 January 2024 (NCT05367440, AstraZeneca, Cambridge, United Kingdom). The study is estimated to enroll 72 participants (age 18–130) and consists of three individual study arms, each group receiving AZD5305 in combination with a hormonal agent. More detailed information is available at http://clinicaltrials.gov. Beyond that, researchers are continuing to test AZD5305 in PARP inhibitor–naïve patients and testing it with the HER2 antibody drug–conjugate (ADC) trastuzumab deruxtecan (Enhertu; AstraZeneca) and the TROP2-directed ADC datopotamab deruxtecan (AstraZeneca/Daiichi Sankyo) (AZD5305 More Tolerable than Earlier, 2022).

### 4.7 Future promise

In conclusion, AZD5305 is a highly potent next-generation PARP inhibitor (PARPi) and trapper, with enhanced anticancer efficacy and improved safety profile in preclinical models compared to other PARPis. Thus, it is an exciting and unique clinical candidate, with a variety of clinical options as a monotherapy or in combination with other agents. Future research should focus on evaluating the side effects, confirming the width of the therapeutic window, selecting eligible human subjects, managing resistance to combined therapies, and decreasing the preparation cost. If the abovementioned issues can be addressed, we firmly believe that a significant proportion of cancer patients will show improved overall survival with the use of AZD5305.

## 5 Conclusion

PARPis have transformed the oncology landscape. Approved PARPis (olaparib, rucaparib, niraparib, and talazoparib) have shown varying levels of selectivity for PARP1 over PARP2 and for the entire PARP family. The current approved PARPi has shown both statistically and clinically significant benefits in the treatment of cancer. Moreover, the application of PARPis in the treatment of non-BRCAm cancer is expected. The irreplaceable function of PARP1 in antitumor activity has led to an interest in the development of next-generation PARPis with improved selectivity for PARP1. AZD5305 was discovered by profiling current clinical and late-stage PARPis using fluorescence polarization binding assays. The discovery of AZD5305 represents a milestone for expanding the potential therapeutic opportunities for PARPis, as monotherapy or in combination with other agents. However, a thorough knowledge of the mechanism of action, indications, safety profile, and rational combination is imperative for the safe use of AZD5305 in clinical applications. Furthermore, the development of reliable methods for the detection of homologous recombination deficiency status is urgently needed to determine the beneficiary population.

## References

[B1] Audeh M. W. Carmichael J. Penson R. T. Friedlander M. Powell B. Bell-McGuinn K. M. (2010). Oral poly(ADP-ribose) polymerase inhibitor olaparib in patients with BRCA1 or BRCA2 mutations and recurrent ovarian cancer: A proof-of-concept trial. Lancet 376, 245–251. 10.1016/s0140-6736(10)60893-8 20609468

[B2] AZD5305 more tolerable than earlier PARP agents. Cancer Discov. 2022;12(7):1602. 10.1158/2159-8290.CD-NB2022-0039 35616497

[B3] Balasubramaniam S. Beaver J. A. Horton S. Fernandes L. L. Tang S. Horne H. N. (2017). FDA approval summary: Rucaparib for the treatment of patients with deleterious BRCA mutation-associated advanced ovarian cancer. Clin. Cancer Res. 23, 7165–7170. 10.1158/1078-0432.Ccr-17-1337 28751443

[B4] Berek J. S. Matulonis U. A. Peen U. Ghatage P. Mahner S. Redondo A. (2018). Safety and dose modification for patients receiving niraparib. Ann. Oncol. 29, 1784–1792. 10.1093/annonc/mdy181 29767688

[B5] Boussios S. Rassy E. Shah S. Ioannidou E. Sheriff M. Pavlidis N. (2021). Aberrations of DNA repair pathways in prostate cancer: A cornerstone of precision oncology. Expert Opin. Ther. Targets 25, 329–333. 10.1080/14728222.2021.1951226 34225539

[B6] Bryant H. E. Schultz N. Thomas H. D. Parker K. M. Flower D. Lopez E. (2005). Specific killing of BRCA2-deficient tumours with inhibitors of poly(ADP-ribose) polymerase. Nature 434, 913–917. 10.1038/nature03443 15829966

[B7] Coleman R. L. Oza A. M. Lorusso D. Aghajanian C. Oaknin A. Dean A. (2017). Rucaparib maintenance treatment for recurrent ovarian carcinoma after response to platinum therapy (ARIEL3): A randomised, double-blind, placebo-controlled, phase 3 trial. Lancet 390, 1949–1961. 10.1016/s0140-6736(17)32440-6 28916367 PMC5901715

[B8] Curtin N. J. Szabo C. (2020). Poly(ADP-ribose) polymerase inhibition: Past, present and future. Nat. Rev. Drug Discov. 19, 711–736. 10.1038/s41573-020-0076-6 32884152

[B9] Dellavedova G. Decio A. Anna S. Leo E. Giavazzi R. Rosa Bani M. (2021). Abstract P217: The next generation PARP inhibitor AZD5305 is active in a broad range of pre-clinical models of ovarian cancer. Mol. Cancer Ther. 20, P217. 10.1158/1535-7163.TARG-21-P217

[B10] Farmer H. McCabe N. Lord C. J. Tutt A. N. Johnson D. A. Richardson T. B. (2005). Targeting the DNA repair defect in BRCA mutant cells as a therapeutic strategy. Nature 434, 917–921. 10.1038/nature03445 15829967

[B11] Farrés J. Llacuna L. Martin-Caballero J. Martínez C. Lozano J. J. Ampurdanés C. (2015). PARP-2 sustains erythropoiesis in mice by limiting replicative stress in erythroid progenitors. Cell Death Differ. 22, 1144–1157. 10.1038/cdd.2014.202 25501596 PMC4568570

[B12] Fong P. C. Yap T. A. Boss D. S. Carden C. P. Mergui-Roelvink M. Gourley C. (2010). Poly(ADP)-ribose polymerase inhibition: Frequent durable responses in BRCA carrier ovarian cancer correlating with platinum-free interval. J. Clin. Oncol. 28, 2512–2519. 10.1200/jco.2009.26.9589 20406929

[B13] Gao Q. Zhu J. Zhao W. Huang Y. An R. Zheng H. (2022). Olaparib maintenance monotherapy in asian patients with platinum-sensitive relapsed ovarian cancer: Phase III trial (L-MOCA). Clin. Cancer Res. 28, 2278–2285. 10.1158/1078-0432.Ccr-21-3023 35131903 PMC9359747

[B14] Gill Sonja J. Macdonald R. Pin C. Maglennon G. (2021). Abstract 1374: The novel PARP1-selective inhibitor AZD5305 has reduced hematological toxicity when compared to PARP1/2 inhibitors in pre-clinical models. Cancer Res. 81, 1374. 10.1158/1538-7445.AM2021-1374

[B15] Gong H. Nie D. Huang Y. Li Z. (2020). Poly (ADP-ribose) polymerase (PARP) inhibitor regimens for ovarian cancer in phase III randomized controlled trials: A network meta-analysis. Int. J. Gynecol. Cancer 30, 1576–1582. 10.1136/ijgc-2020-001373 32817083

[B16] Gong H. Nie D. Li Z. (2020). Targeting six hallmarks of cancer in ovarian cancer therapy. Curr. Cancer Drug Targets 20, 853–867. 10.2174/1568009620999200816130218 32807056

[B17] González-Martín A. Pothuri B. Vergote I. DePont Christensen R. Graybill W. Mirza M. R. (2019). Niraparib in patients with newly diagnosed advanced ovarian cancer. N. Engl. J. Med. 381, 2391–2402. 10.1056/NEJMoa1910962 31562799

[B18] Hande S. Balazs A. Degorce S. L. Embrey K. Ghosh A. Gill S. J. (2021). Abstract 296: Structure-based and property-based drug design of AZD5305, a highly selective PARP1 inhibitor and trapper. Proc. Cancer Chem. 81, 296. 10.1158/1538-7445.am2021-296

[B19] Hao J. Liu Y. Zhang T. He J. Zhao H. An R. (2021). Efficacy and safety of PARP inhibitors in the treatment of advanced ovarian cancer: An updated systematic review and meta-analysis of randomized controlled trials. Crit. Rev. Oncol. Hematol. 157, 103145. 10.1016/j.critrevonc.2020.103145 33254040

[B20] Hoy S. M. (2018). Talazoparib: First global approval. Drugs 78, 1939–1946. 10.1007/s40265-018-1026-z 30506138

[B21] Illuzzi G. Staniszewska A. D. Gill S. J. Pike A. McWilliams L. Critchlow S. E. (2022). Preclinical characterization of AZD5305, a next generation, highly selective PARP1 inhibitor and trapper. Clin. Cancer Res. 28, 4724–4736. 10.1158/1078-0432.CCR-22-0301 35929986 PMC9623235

[B22] Illuzzi G. McWilliams L. Jamal K. Galbiati A. Bentouati S. Griffiths D. (2021). “Abstract 1272:In vitrocellular profiling of AZD5305, novel PARP1-selective inhibitor and trapper,” in Proceedings of the experimental and molecular therapeutics, 1272.

[B23] Ison G. Howie L. J. Amiri-Kordestani L. Zhang L. Tang S. Sridhara R. (2018). FDA approval summary: Niraparib for the maintenance treatment of patients with recurrent ovarian cancer in response to platinum-based chemotherapy. Clin. Cancer Res. 24, 4066–4071. 10.1158/1078-0432.Ccr-18-0042 29650751

[B24] Johannes J. W. Balazs A. Barratt D. Bista M. Chuba M. D. Cosulich S. (2021). Discovery of 5-{4-[(7-Ethyl-6-oxo-5, 6-dihydro-1, 5-naphthyridin-3-yl)methyl]piperazin-1-yl}-N-methylpyridine-2-carboxamide (AZD5305): A PARP1-DNA trapper with high selectivity for PARP1 over PARP2 and other PARPs. J. Med. Chem. 64, 14498–14512. 10.1021/acs.jmedchem.1c01012 34570508

[B25] Kedar P. S. Stefanick D. F. Horton J. K. Wilson S. H. (2012). Increased PARP-1 association with DNA in alkylation damaged, PARP-inhibited mouse fibroblasts. Mol. Cancer Res. 10, 360–368. 10.1158/1541-7786.Mcr-11-0477 22246237 PMC3307909

[B26] Kim G. Ison G. McKee A. E. Zhang H. Tang S. Gwise T. (2015). FDA approval summary: Olaparib monotherapy in patients with deleterious germline BRCA-mutated advanced ovarian cancer treated with three or more lines of chemotherapy. Clin. Cancer Res. 21, 4257–4261. 10.1158/1078-0432.Ccr-15-0887 26187614

[B27] LaFargue C. J. Dal Molin G. Z. Sood A. K. Coleman R. L. (2019). Exploring and comparing adverse events between PARP inhibitors. Lancet Oncol. 20, e15–e28. 10.1016/s1470-2045(18)30786-1 30614472 PMC7292736

[B28] Leo E. Johannes J. (2021). “Abstract ND05: Discovery and first structural disclosure of AZD5305: A next generation, highly selective PARP1 inhibitor and trapper,” in Proceedings of the experimental and molecular therapeutics, ND05.

[B29] Li H. Liu Z. Y. Wu N. Chen Y. C. Cheng Q. Wang J. (2020). PARP inhibitor resistance: The underlying mechanisms and clinical implications. Mol. Cancer 19, 107. 10.1186/s12943-020-01227-0 32563252 PMC7305609

[B30] Litton J. K. Rugo H. S. Ettl J. Hurvitz S. A. Gonçalves A. Lee K. H. (2018). Talazoparib in patients with advanced breast cancer and a germline BRCA mutation. N. Engl. J. Med. 379 (8), 753–763. 10.1056/NEJMoa1802905 30110579 PMC10600918

[B31] LYNPARZA® (olaparib). U.S (2019). Food and drug administration website. Available at:https://www.accessdata.fda.gov/drugsatfda_docs/label/2018/208558s001lbl.pdf (Accessed on Dec 20, 2019).

[B32] Madariaga A. Bowering V. Ahrari S. Oza A. M. Lheureux S. (2020). Manage wisely: Poly (ADP-ribose) polymerase inhibitor (PARPi) treatment and adverse events. Int. J. Gynecol. Cancer 30 (7), 903–915. 10.1136/ijgc-2020-001288 32276934 PMC7398227

[B33] Mirza M. R. Monk B. J. Herrstedt J. Oza A. M. Mahner S. Redondo A. (2016). Niraparib maintenance therapy in platinum-sensitive, recurrent ovarian cancer. N. Engl. J. Med. 375, 2154–2164. 10.1056/NEJMoa1611310 27717299

[B34] Moore K. Colombo N. Scambia G. Kim B. G. Oaknin A. Friedlander M. (2018). Maintenance olaparib in patients with newly diagnosed advanced ovarian cancer. N. Engl. J. Med. 379, 2495–2505. 10.1056/NEJMoa1810858 30345884

[B35] Murai J. Huang S. Y. Das B. B. Renaud A. Zhang Y. Doroshow J. H. (2012). Trapping of PARP1 and PARP2 by clinical PARP inhibitors. Cancer Res. 72, 5588–5599. 10.1158/0008-5472.Can-12-2753 23118055 PMC3528345

[B36] Murai J. Zhang Y. Morris J. Ji J. Takeda S. Doroshow J. H. (2014). Rationale for poly(ADP-ribose) polymerase (PARP) inhibitors in combination therapy with camptothecins or temozolomide based on PARP trapping versus catalytic inhibition. J. Pharmacol. Exp. Ther. 349, 408–416. 10.1124/jpet.113.210146 24650937 PMC4019318

[B37] National Comprehensive Cancer Network (NCCN) (2019). NCCN clinical practice guidelines in oncology. Palliative care. Version 2. Available at:https://www.nccn.org/professionals/physician_gls/pdf/palliative.pdf . 10.6004/jnccn.2009.0031 19406043

[B38] Nishizuka Y. Ueda K. Nakazawa K. Hayaishi O. (1967). Studies on the polymer of adenosine diphosphate ribose. J. Biol. Chem. 242, 3164–3171. 10.1016/s0021-9258(18)95947-8 4291072

[B39] Oza A. M. Matulonis U. A. Malander S. Hudgens S. Sehouli J. Del Campo J. M. (2018). Quality of life in patients with recurrent ovarian cancer treated with niraparib versus placebo (ENGOT-OV16/NOVA): Results from a double-blind, phase 3, randomised controlled trial. Lancet Oncol. 19, 1117–1125. 10.1016/S1470-2045(18)30333-4 30026000

[B40] Papeo G. Posteri H. Borghi D. Busel A. A. Caprera F. Casale E. (2015). Discovery of 2-[1-(4, 4-Difluorocyclohexyl)piperidin-4-yl]-6-fluoro-3-oxo-2, 3-dihydro-1H-isoindole-4-carboxamide (nms-P118): A potent, orally available, and highly selective PARP-1 inhibitor for cancer therapy. J. Med. Chem. 58, 6875–6898. 10.1021/acs.jmedchem.5b00680 26222319

[B41] Pilie P. G. Gay C. M. Byers L. A. O’Connor M. J. Yap T. A. (2019). PARP inhibitors: Extending benefit beyond BRCA-mutant cancers. Clin. Cancer Res. 25, 3759–3771. 10.1158/1078-0432.CCR-18-0968 30760478

[B42] Pommier Y. O'Connor M. J. de Bono J. (2016). Laying a trap to kill cancer cells: PARP inhibitors and their mechanisms of action. Sci. Transl. Med. 8, 362ps17. 10.1126/scitranslmed.aaf9246 27797957

[B43] Poveda A. Lheureux S. Colombo N. Cibula D. Lindemann K. Weberpals J. (2022). Olaparib maintenance monotherapy in platinum-sensitive relapsed ovarian cancer patients without a germline BRCA1/BRCA2 mutation: OPINION primary analysis. Gynecol. Oncol. 164, 498–504. 10.1016/j.ygyno.2021.12.025 35063276

[B44] Pujade-Lauraine E. Ledermann J. A. Selle F. Gebski V. Penson R. T. Oza A. M. (2017). Olaparib tablets as maintenance therapy in patients with platinum-sensitive, relapsed ovarian cancer and a BRCA1/2 mutation (SOLO2/ENGOT-Ov21): A double-blind, randomised, placebo-controlled, phase 3 trial. Lancet Oncol. 18, 1274–1284. 10.1016/s1470-2045(17)30469-2 28754483

[B45] Ray-Coquard I. Pautier P. Pignata S. Pérol D. González-Martín A. Berger R. (2019). Olaparib plus bevacizumab as first-line maintenance in ovarian cancer. N. Engl. J. Med. 381, 2416–2428. 10.1056/NEJMoa1911361 31851799

[B46] Reiss K. A. Mick R. Teitelbaum U. O'Hara M. Schneider C. Massa R. (2022). Niraparib plus nivolumab or niraparib plus ipilimumab in patients with platinum-sensitive advanced pancreatic cancer: A randomised, phase 1b/2 trial. Lancet Oncol. 23 (8), 1009–1020. 10.1016/S1470-2045(22)00369-2 35810751 PMC9339497

[B47] Robson M. Im S. A. Senkus E. Xu B. Domchek S. M. Masuda N. (2017). Olaparib for metastatic breast cancer in patients with a germline BRCA Mutation. Erratum in. N. Engl. J. MedN Engl. J. Med. 377 (6), 523–533. 10.1056/NEJMoa1706450 28578601

[B48] Ronson G. E. Piberger A. L. Higgs M. R. Olsen A. L. Stewart G. S. McHugh P. J. (2018). PARP1 and PARP2 stabilise replication forks at base excision repair intermediates through Fbh1-dependent Rad51 regulation. Nat. Commun. 9, 746. 10.1038/s41467-018-03159-2 29467415 PMC5821833

[B49] RUBRACA® (rucaparib) tablets (2019). U.S. Food and Drug Administration website. Available:https://www.accessdata.fda.gov/drugsatfda_docs/label/2018/209115s003lbl.pdf (Accessed on Dec 20, 2019).

[B50] Staniszewska A. D. Jwt J. W. Y. Pike A. Fazenbaker C. Cook K. Bosco E. (2021). “Abstract 1270: The novel PARP1-selective inhibitor, AZD5305, is efficacious as monotherapy and in combination with standard of care chemotherapy in thein vivopreclinical models,” in Proceedings of the experimental and molecular therapeutics, 1270.

[B51] Sun W. Li J. Zhang Z. Su X. (2021). Gastrointestinal events with PARP inhibitors in cancer patients: A meta-analysis of phase II/III randomized controlled trials. J. Clin. Pharm. Ther. 46, 241–255. 10.1111/jcpt.13300 33135237

[B52] Tung N. M. Robson M. E. Ventz S. Santa-Maria C. A. Nanda R. Marcom P. K. (2020). Tbcrc 048: Phase II study of olaparib for metastatic breast cancer and mutations in homologous recombination-related genes. J. Clin. Oncol. 38 (36), 4274–4282. 10.1200/JCO.20.02151 33119476

[B53] Tutt A. N. J. Garber J. E. Kaufman B. Viale G. Fumagalli D. Rastogi P. (2021). Adjuvant olaparib for patients with *BRCA1*- or *BRCA2*-mutated breast cancer. N. Engl. J. Med. 384 (25), 2394–2405. 10.1056/NEJMoa2105215 34081848 PMC9126186

[B54] Xu Q. Li Z. (2021). Update on poly ADP-ribose polymerase inhibitors in ovarian cancer with non-BRCA mutations. Front. Pharmacol. 12, 743073. 10.3389/fphar.2021.743073 34912215 PMC8667582

[B55] Zandarashvili L. Langelier M. F. Velagapudi U. K. Hancock M. A. Steffen J. D. Billur R. (2020). Structural basis for allosteric PARP-1 retention on DNA breaks. Science 368, eaax6367. 10.1126/science.aax6367 32241924 PMC7347020

[B56] ZEJULATM (niraparib) capsules (2019). U.S. Food and Drug Administration website. Available at:https://www.accessdata.fda.gov/drugsatfda_docs/label/2017/208447lbl.pdf (Accessed on Dec 20, 2019).

