# Supplementary material for: Current status and future promise of next-generation poly (ADP-Ribose) polymerase 1-selective inhibitor AZD5305
Source: Front Pharmacol. 2023 Jan 23;13:979873. doi: 10.3389/fphar.2022.979873 (PMC9899804; doi:10.3389/fphar.2022.979873)
Supplement: Supplementary file 1 [file DataSheet1.PDF]

Supplementary Material

Table 1. Summary of adverse events in phase Ⅲ trials of PARPi in the front-line ovarian cancer setting

	**Olaparib**												**Rucaparib**				**Niraparib**							
	SOLO1				SOLO2/ENGOT-Ov21				PAOLA-1				ARIEL3				ENGOT-OV16/NOVA				PRIMA/ENGOT-OV26 /GOG-3012			
	Olaparib (n=260)		Placebo (n=130)		Olaparib (n=195)		Placebo (n=99)		Olaparib plus Bevacizumab (n=535)		Placebo plus Bevacizumab (n=267)		Rucaparib (n=372)		Placebo (n=189)		Niraparib (n=367)		Placebo (n=179)		Niraparib (n=484)		Placebo (n=244)	
	Any grade	Grade 3 or 4	Any grade	Grade 3 or 4	Any grade	Grade 3 or 4	Any grade	Grade 3 or 4	Any grade	Grade 3 or 4	Any grade	Grade 3 or 4	Any grade	Grade 3 or 4	Any grade	Grade 3 or 4	Any grade	Grade 3 or 4	Any grade	Grade 3 or 4	Any grade	Grade 3 or 4	Any grade	Grade 3 or 4
	Number of patients (percent)																							
**Hematological toxicities**																								
**Anemia**	101 (39)	56 (22)	13 (10)	2 (2)	85 (44)	38 (19)	8 (8)	2 (2)	219 (41)	93 (17)	27 (10)	1 (<1)	139 (37)	70 (19)	11 (6)	1 (1)	184 (50)	93 (25)	12 (7)	0	307 (63)	150 (31)	43 (18)	4 (2)
**Thrombocytopenia**	29 (11)	2 (1)	5 (4)	2 (2)	27 (14)	2 (1)	3 (3)	1 (1)	42 (8)	9 (2)	9 (3)	1 (<1)	104 (28)	13 (5)	5 (3)	0	225 (61)	124 (34)	10 (6)	1(<1)	222 (46)	139 (29)	9 (4)	1 (<1)
**Neutropenia**	60 (23)	22 (9)	15 (12)	6 (5)	38 (19)	10 (5)	6 (6)	4 (4)	95 (18)	32 (6)	42 (16)	8 (3)	67 (18)	25 (7)	9 (5)	2 (1)	111 (30)	72 (20)	11 (6)	3 (2)	128 (26)	62 (13)	16 (7)	3 (1)
**Lymphopenia**	NR	NR	NR	NR	NR	NR	NR	NR	126 (24)	38 (7)	25 (9)	3 (1)	NR	NR	NR	NR	NR	NR	NR	NR	NR	NR	NR	NR

**Leukopenia**	NR	NR	NR	NR	20 (10)	3 (2)	1 (1)	0	95 (18)	10 (2)	26 (10)	4 (1)	NR	NR	NR	NR	NR	NR	NR	NR	NR	NR	NR	NR
**Gastrointestinal toxicities**																								
**Nausea**	201 (77)	2 (1)	49 (38)	0	148 (76)	5 (3)	33 (33)	0	285 (53)	13 (2)	58 (22)	2 (1)	280 (75)	14 (4)	69 (37)	1 (1)	270 (74)	11 (3)	63 (35)	2 (1)	278 (57)	6 (1)	67 (28)	2 (<1)
**Constipation**	72 (28)	0	25 (19)	0	40 (21)	0	23 (23)	3 (3)	53 (10)	0	28 (10)	1 (<1)	136 (37)	7 (2)	45 (24)	2 (1)	146 (40)	2(<1)	36 (20)	1 (<1)	189 (39)	1 (<1)	46 (19)	0
**Vomiting**	104 (40)	1 (<1)	19 (15)	1 (1)	73 (37)	5 (3)	19 (19)	1 (1)	117 (22)	8 (1)	29 (11)	5 (2)	136 (37)	15 (4)	28 (15)	2 (1)	126 (34)	7 (2)	29 (16)	1 (<1)	108 (22)	4 (<1)	29 (12)	2 (<1)
**Decreased appetite**	51 (20)	0	13 (10)	0	43 (22)	0	11 (11)	0	NR	NR	NR	NR	87 (23)	2 (1)	26 (14)	0	93 (25)	1 (<1)	26 (15)	1 (<1)	NR	NR	NR	NR
**Abdominal pain**	64 (25)	4 (2)	25 (19)	1 (1)	47 (24)	5 (3)	31 (31)	3 (3)	103 (19)	8 (1)	53 (20)	5 (2)	111 (30)	9 (2)	49 (26)	1 (1)	83 (23)	4 (1)	53 (30)	3 (2)	106 (22)	7 (2)	75 (31)	1 (<1)
**Upper abdominal pain**	46 (18)	0	17 (13)	0	21 (11)	0	12 (12)	0	NR	NR	NR	NR	52 (14)	2 (1)	10 (5)	0	NR	NR	NR	NR	NR	NR	NR	NR
**Diarrhea**	89 (34)	8 (3)	32 (25)	0	64 (33)	2 (1)	20 (20)	0	98 (18)	12 (2)	45 (17)	5 (2)	118 (32)	2 (1)	41 (22)	2 (1)	70 (19)	1 (<1)	37 (21)	2 (1)	NR	NR	NR	NR
**Dyspepsia**	43 (17)	0	16 (12)	0	22 (11)	0	8 (8)	0	NR	NR	NR	NR	54 (15)	1 (<1)	9 (5)	0	42 (11)	0	17 (10)	0	NR	NR	NR	NR
**Abdominal distention**	NR	NR	NR	NR	NR	NR	NR	NR	NR	NR	NR	NR	41 (11)	0	22 (12)	0	28 (8)	0	22 (12)	1 (<1)	NR	NR	NR	NR
**Renal toxicities**																								
**Urinary tract**	NR	NR	NR	NR	18(9	1 (1)	10	0	79	1	27	1	NR	NR	NR	NR	38	3	11	2 (1)	NR	NR	NR	NR

**infection**					)		(10)		(15)	(<1)	(10)	(<1)					(10)	(<1)	(6)					
**Proteinuria**	NR	NR	NR	NR	NR	NR	NR	NR	31 (6)	5 (1)	40 (15)	1 (<1)	NR	NR	NR	NR	NR	NR	NR	NR	NR	NR	NR	NR
**General toxicities**																								
**Fatigue/ asthenia**	165 (63)	10 (4)	54 (42)	2 (2)	128 (66)	8 (4)	39 (39)	2 (2)	283 (53)	28 (5)	86 (32)	4 (1)	258 (69)	25 (7)	83 (44)	5 (3)	218 (59)	30 (8)	74 (41)	1(<1)	168 (35)	9 (2)	72 (30)	1(<1)
**Pyrexia**	NR	NR	NR	NR	26 (13)	0	6 (6)	0	NR	NR	NR	NR	44 (12)	0	8 (4)	0	NR	NR	NR	NR	NR	NR	NR	NR
**Nervous system toxicities**																								
**Dizziness**	51 (20)	0	20 (15)	1 (<1)	26 (13)	1 (1)	5 (5)	0	NR	NR	NR	NR	54 (15)	0	15 (8)	1 (1)	61 (17)	0	13 (7)	0	NR	NR	NR	NR
**Headache**	59 (23)	1 (<1)	31 (24)	3 (2)	49 (25)	1 (1)	13 (13)	0	73 (14)	2 (<1)	36 (13)	2 (1)	67 (18)	1 (<1)	30 (16)	1 (1)	95 (26)	1 (<1)	17 (10)	0	126 (26)	2 (<1)	36 (15)	0
**Dysgeusia**	68 (26)	0	5 (4)	0	52 (27)	0	7 (7)	0	NR	NR	NR	NR	146 (39)	0	13 (7)	0	37 (10)	0	7 (4)	0	NR	NR	NR	NR
**Respiratory toxicities**																								
**Dyspnea**	39 (15)	0	7 (5)	0	23 (12)	1 (1)	0	0	NR	NR	NR	NR	50 (13)	0	14 (7)	0	71 (19)	4 (1)	15 (8)	2 (1)	NR	NR	NR	NR
**Nasopharyngitis**	NR	NR	NR	NR	21 (11)	0	11 (11)	0	NR	NR	NR	NR	NR	NR	NR	NR	41 (11)	0	13 (7)	0	NR	NR	NR	NR
**Cough**	42 (16)	0	28 (22)	0	33 (17)	1 (1)	5 (5)	0	NR	NR	NR	NR	54 (15)	0	25 (13)	0	55 (15)	0	8 (5)	0	NR	NR	NR	NR
**Psychiatric toxicities**																								

**Insomnia**	NR	NR	NR	NR	NR	NR	NR	NR	NR	NR	NR	NR	53 (14)	0	15 (8)	0	89 (24)	1 (<1)	13 (7)	0	119 (25)	4 (<1)	35 (14)	1 (<1)
**Musculoskeletal and connective tissue toxicities**																								
**Arthralgia**	66 (25)	0	35 (27)	0	29 (15)	0	15 (15)	0	116 (22)	3 (1)	64 (24)	4 (1)	57 (15)	2 (1)	24 (13)	0	43 (12)	1 (<1)	22 (12)	0	NR	NR	NR	NR
**Back pain**	40 (15)	0	16 (12)	0	22 (11)	0	13 (13)	2 (2)	NR	NR	NR	NR	45 (12)	0	28 (15)	0	49 (13)	2 (<1)	21 (12)	0	NR	NR	NR	NR
**Myalgia**	NR	NR	NR	NR	NR	NR	NR	NR	NR	NR	NR	NR	NR	NR	NR	NR	30 (8)	1 (<1)	18 (10)	0	NR	NR	NR	NR
**Cardiovascular toxicities**																								
**Hypertension**	NR	NR	NR	NR	NR	NR	NR	NR	245 (46)	100 (19)	160 (60)	81 (30)	NR	NR	NR	NR	71 (19)	30 (8)	8 (5)	4 (2)	NR	NR	NR	NR
**Palpitations**	NR	NR	NR	NR	NR	NR	NR	NR	NR	NR	NR	NR	NR	NR	NR	NR	38 (10)	0	3 (2)	0	NR	NR	NR	NR
**AE leading to dose interruption**	135 (52)	NA	22 (17)	NA	88 (45)	NA	18 (18)	NA	291 (54)	NA	65 (24)	NA	237 (64)	NA	19 (10)	NA	253 (69)	NA	9 (5)	NA	385 (80)	NA	44 (18)	NA
**AE leading to dose reduction**	74 (28)	NA	4 (3)	NA	49 (25)	NA	3 (3)	NA	220 (41)	NA	20 (7)	NA	203 (55)	NA	8 (4)	NA	244 (67)	NA	26 (15)	NA	343 (71)	NA	20 (8)	NA
**AE leading to discontinuation of intervention**	30 (12)	NA	3 (2)	NA	21 (11)	NA	2 (2)	NA	109 (20)	NA	15 (6)	NA	263 (71)	NA	20 (11)	NA	54 (15)	NA	4 (2)	NA	58 (12)	NA	6 (3)	NA

AE, adverse event; NA, not available; NR, not reported; PARPi, poly (ADP-ribose) polymerase inhibitor.

Table 2. Summary of adverse events in phase Ⅲ trials of PARPi in the front-line breast cancer setting

	Olaparib								Talazoparib			
	OlympiA trial				OlympiAD trial				EMBRACA trial			
	Olaparib(N=911)		Placebo(N=904)		Olaparib(N=205)		Standard-Therapy(N=91)		Talazoparib(N=286)		Standard-Therapy(N=126)	
	Any Grade	Grade ≥3	Any Grade	Grade ≥3	Any Grade	Grade ≥3	Any Grade	Grade ≥3	Any Grade	Grade ≥3	Any Grade	Grade ≥3
	Number of patients (percent)											
**Anemia**	214(23.5)	79(8.7)	35(3.9)	3(0.3)	82(40.0)	33(16.1)	24(26.4)	4(4.4)	151(52.8)	112(39.2)	23(18.3)	6(4.8)
**Decreased white-cell count**	143(15.7)	27(3.0)	52(5.8)	3(0.3)	33(16.1)	7(3.4)	19(20.9)	9(9.9)	49(17.1)	19(6.6)	17(13.5)	11(8.7)
**Nausea**	518(56.9)	7(0.8)	211(23.3)	0	119(58.0)	0	32(35.2)	1(1.1)	139(48.6)	1(0.3)	59(46.8)	2(1.6)
**Fatigue**	365(40.1)	16(1.8)	245(27.1)	4(0.4)	59(28.8)	6(2.9)	21(23.1)	1(1.1)	144(50.3)	5(1.7)	54(42.9)	4(3.2)
**Vomiting**	206(22.6)	6(0.7)	74(8.2)	0	61(29.8)	0	14(15.4)	1(1.1)	71(24.8)	7(2.4)	29(23.0)	2(1.6)
**Diarrhea**	160(17.6)	3(0.3)	124(13.7)	3(0.3)	42(20.5)	1(0.5)	20(22.0)	0	63(22.0)	2(0.7)	33(26.2)	7(5.6)
**Decreased appetite**	119(13.1)	2(0.2)	53(5.9)	0	33(16.1)	0	11(12.1)	0	61(21.3)	1(0.3)	28(22.2)	1(0.8)

Table 3. Overview of current ongoing clinical trials investigating AZD5305

**Drug**	**AZD5305**	
**NCT Number**	NCT05367440	NCT04644068
**Study Type**	Interventional	Interventional
**Study Phase**	Phase I/IIa	Phase I/IIa
**Estimated/Actual Enrollment**	72	715
**Brief Summary**	This is a multi-arm, open-label phase I/IIa study to assess the safety, tolerability, phamacokinetics, pharmacodynamics and preliminary efficacy of AZD5305 in combination with new hormonal agents with metastatic prostate cancer	This is a phase I/IIa modular, open-label, multi-center study of AZD5305 administered orally, either as monotherapy or in combination with other anti-cancer agents in patients with advanced solid malignancies to determine if experimental treatment with PARP inhibitor AZD5305, alone or in combination with anti-cancer agents is safe, tolerable, and has anti-cancer activity in patients with advanced solid tumors
**Study Design**	Allocation: non-randomized<br>Intervention Model: parallel assignment<br>Intervention Model Description:<br>● Arm 1 (AZD5305 + enzalutamide)<br>● Arm 2 (AZD5305 + abiraterone acetate)<br>● Arm 3 (AZD5305 + darolutamide)<br>Masking: none (open label)<br>Primary Purpose: treatment	Allocation: non-randomized<br>Intervention Model: sequential assignment<br>Intervention Model Description:<br>● Module 1 (AZD5305 monotherapy)<br>● Module 2 (AZD5305 + paclitaxel)<br>● Module 3 (AZD5305 + carboplatin, +/- paclitaxel)<br>● Module 4 (AZD5305 + T DXd)<br>● Module 5 (AZD5305 + Dato-DXd)<br>Each module has 2 study parts:<br>Part A consisting of dose-escalation cohorts<br>Part B consisting of expansion cohorts

		Masking: none (open label)<br><br>Primary Purpose: treatment
**Disease/Condition**	Metastatic prostate Cancer	Ovarian Cancer, Breast Cancer, Pancreatic Cancer, Prostate Cancer<br><br>Additional Indications Below for Module 4 and 5<br><br>Non-small Cell Lung Cancer, Small Cell Lung Cancer, Colorectal Cancer, Bladder Cancer, Gastric Cancer, Biliary Cancer, Cervical Cancer, Endometrial Cancer
**Listed Location Countries**	Australia, Italy, United Kingdom	Australia, Canada, China, Czechia, Hungary, Italy, Japan, Korea, Poland, Russian Federation, Spain, United Kingdom, United States
**Study Status**	Recruiting	Recruiting
**Actual Start Date**	June 2, 2022	November 12, 2020
**Estimated Completion Date**	January 12, 2024	July 29, 2025
**Study Sponsor**	AstraZeneca	AstraZeneca
